# Isolated Persistent Left Superior Vena Cava Associated With Autosomal Dominant Polycystic Kidney Disease (ADPKD): Challenges and Clinical Significance

**DOI:** 10.7759/cureus.22890

**Published:** 2022-03-06

**Authors:** Osama A Samara, Osama Alzoubi, Ahmad M Gharaibeh, Qusai Alnajjar, Izzat Alawwa

**Affiliations:** 1 Department of Diagnostic and Interventional Radiology, Jordan University Hospital, Amman, JOR; 2 Medical School, University of Jordan, Amman, JOR; 3 Department of Internal Medicine, Jordan University Hospital, Amman, JOR; 4 Department of Nephrology, Jordan University Hospital, Amman, JOR

**Keywords:** 3d-reconstructiton, cardiac mri, adpkd, autosomal dominant polycystic kidney disease, persistent left superior vena cava

## Abstract

Persistent left superior vena cava (PLSVC) is the most common venous anomaly of the thorax that usually coexists with the right superior vena cava. However, in a minority of cases, there is only a PLSVC without a right superior vena cava which is called an isolated PLSVC. There are some reported anomalies that can co-occur with PLSVC; yet, none have reported an association with autosomal dominant polycystic kidney disease (ADPKD). In this case report, we describe a 41-year-old man with end-stage renal disease from ADPKD who underwent central venous catheterization (permacath) to initiate hemodialysis. Upon catheterization, a complete right internal jugular vein septum (bicuspid valve) was found, along with an isolated PLSVC that drained directly in the coronary sinus. We demonstrate the multiple challenges encountered during the catheterization procedure and we illustrate the anomaly with detailed images and supplementary videos. Furthermore, we discuss the coexistence of PLSVC with renal anomalies in the context of previous literature. To conclude, interventional radiologists should keep the possibility of PLSVC in mind, especially when difficulties are encountered during catheterization where proper characterization of the PLSVC type and associated anomalies is crucial for tailoring the best management approach. Moreover, an association between venous anomalies including left superior vena cava and renal anomalies may co-exist, and further studies are needed to explore this possible association.

## Introduction

Persistent left superior vena cava (PLSVC) is the most common venous anomaly of the thorax that usually coexists with the right superior vena cava. Postnatal series and autopsy-based studies have reported an overall incidence of PLSVC of 0.3-0.5% in the general healthy population and of 4-8% in patients with congenital heart disease [1]. This condition results from the failure of the left anterior cardinal vein to degenerate during embryological development [2]. It is usually an incidental finding in central venous catheterization, where the catheter gets directed to the left mediastinum instead of the expected pathway to the right atrium. However, it may be associated with different clinical scenarios, such as arrhythmias, cryptogenic strokes, and difficult heart catheterization [3].

Most commonly, PLSVC coexists with right superior vena cava (80-90% of cases) [4]. In other cases, there is only a persistent left superior vena cava without a normal or aberrant right superior vena cava called an isolated PLSVC. Here, we describe a patient who underwent catheterization of the right internal jugular vein for hemodialysis. He had autosomal dominant polycystic kidney disease (ADPCK). We highlight the importance of always considering possible exposure to such venous anomalies during catheterization and discussing the co-occurrence of isolated PLSVC and ADPKD.

## Case presentation

A 41-year-old man with a medical history significant for end-stage renal disease, old myocardial infarction, heart failure with reduced ejection fraction, deep vein thrombosis, and hypertension was admitted to our institution to initiate hemodialysis. He is a smoker (24 pack-years). The end-stage renal disease was associated with autosomal dominant polycystic kidney disease (ADPKD).

**Figure 1:**
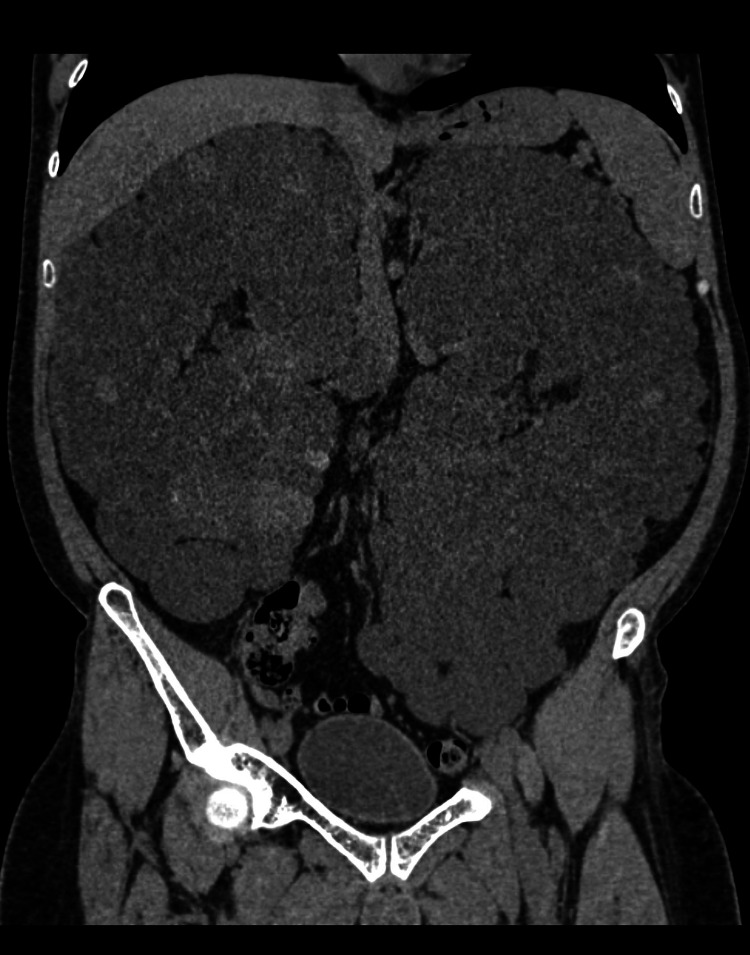
Abdominal CT scan showing markedly enlarged kidneys bilaterally extending downwards to the pelvis with innumerable multiple variable-sized renal cysts. Right kidney measures about 29cm, and the left kidney measures about 33cm.

Four years ago, the patient had an inferior myocardial infarction with subsequent percutaneous transluminal coronary angioplasty of the distal right coronary artery without stent placement. His estimated ejection fraction was 30%, and he complained of dyspnea upon exertion for two years. Also, the patient had left lower limb deep vein thrombosis (DVT) five years ago, a complication of ADPKD, and took warfarin for one year. Hypercoagulability workup was negative. He also complained of abdominal distention accompanied by bloating and constipation. Gastrointestinal (GI) workup was unrevealing with normal upper and lower endoscopy and normal liver function test. GI symptoms were most probably due to the mass effect of the large polycystic kidneys (Figure 1). Thus, the patient was referred to urology for possible reduction nephrectomy.

The patient had monthly appointments at the nephrology clinic for years to follow up on his chronic kidney disease. In the last two visits, he complained of worsening generalized fatigue and malaise in addition to anorexia and indigestion. His eGFR was 15 mL/min/1.73m2 and Cr = 6.7 mg/dL (baseline = 5.0-5.5 mg/dl). Thus, the patient was scheduled to undergo arteriovenous fistula creation for initiation of hemodialysis, was considered a moderate-to-high risk patient according to cardiologic evaluation. Thus, he was planned for permacath insertion instead. His moderate-to-high risk cardiologic evaluation also deemed him unfit for reduction nephrectomy.

Physical examination was remarkable for lower limb edema at admission, and two abdominal masses were noticed on each side. The right mass was slightly larger than the left mass and both slightly tender to palpation. Laboratory findings demonstrated serum sodium of 141 mMol/L, potassium of 5.3 mMol/L, chloride of 108 mMol/L, blood urea nitrogen of 67.2 mg/dl, phosphorus of 4.7mg/dl and creatinine of 6.08 mg/dL. INR was 1.5, and partial thromboplastin time (PTT) was 33.2 seconds. The patient was prepared for permacath insertion by the vascular surgery team. Under ultrasound guidance and aseptic techniques, successful cannulation of the right internal jugular vein (IVC) was done. A wire was passed, but resistance was experienced. Following manipulative techniques, a wire was passed, but it went from the right jugular vein to the left thoracic cavity. The vascular team stopped the procedure and re-scheduled a second trial of permacath insertion by the interventional radiology team.

**Figure 2:**
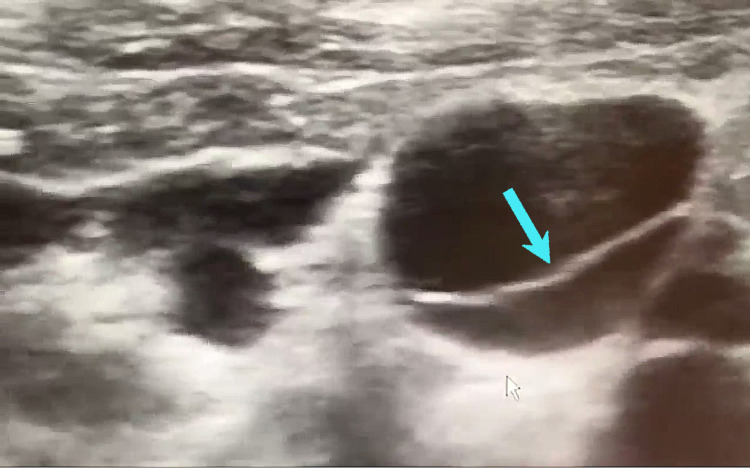
Axial greyscale Right internal jugular vein ultrasound image. Axial greyscale right internal jugular vein ultrasound image showing a right internal jugular vein complete valve as a thin echogenic linear structure in the central lumen at the level of the thoracic inlet (arrow). The valve is of the bicuspid type, in which its motion throughout the cardiac cycle can be appreciated, as seen in Video 1.

Before the second trial of permacath insertion, preliminary ultrasound was done for the jugular veins and showed a complete valve (septum) at the level of the thoracic inlet in both the right and left internal jugular veins (Figure 2). The valve is of the bicuspid type, in which its motion throughout the cardiac cycle can be appreciated, as seen in (Video 1) [5]. Successful cannulation of the right IJV was done after the valve was punctured, serially dilated, and split.

**Video 1:** Right internal jugular vein ultrasound video showing a right internal jugular vein complete bicuspid valve as a thin echogenic linear structure in the central lumen at the level of the thoracic inlet. Notice the valvular motion during the cardiac cycle as the valve closure occurs during diastole (when the atrium transmits backward pressure from the right atrium into the SVC and then into the IJV [5]), but the valve is then open in mid-systole.

**Figure 3:**
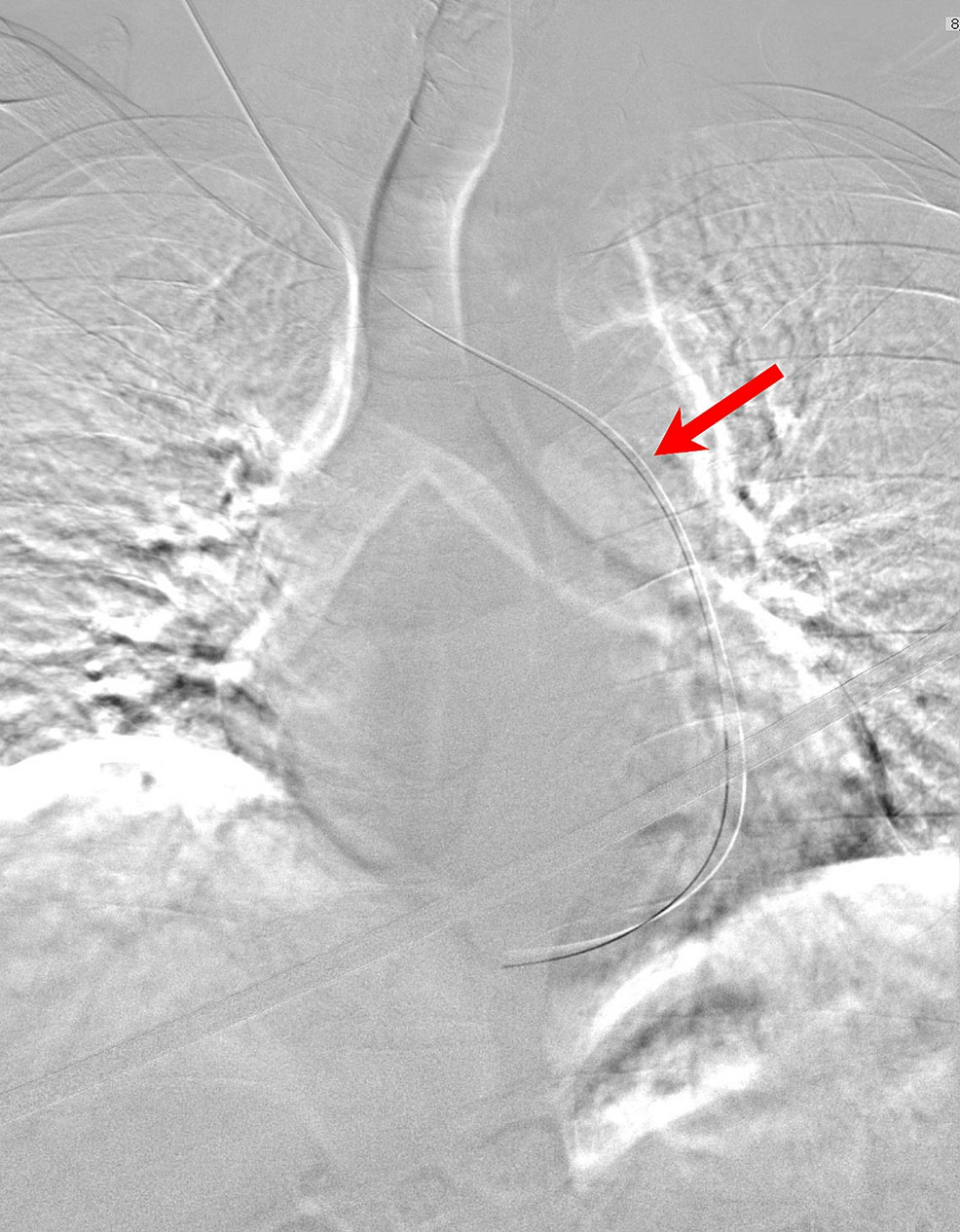
Digital subtraction fluoroscopic image during cannulation of the right IJV. Digital subtraction fluoroscopic image during cannulation of the right IJV showing the guidewire (Arrow) passing to the left thoracic cavity instead of the normal route directly towards the right atrium. After contrast venography and 3d-reconstruction, it was evident that the guidewire traveled through the right brachiocephalic vein, which is directly connected with the isolated PLSVC.

**Figure 4:**
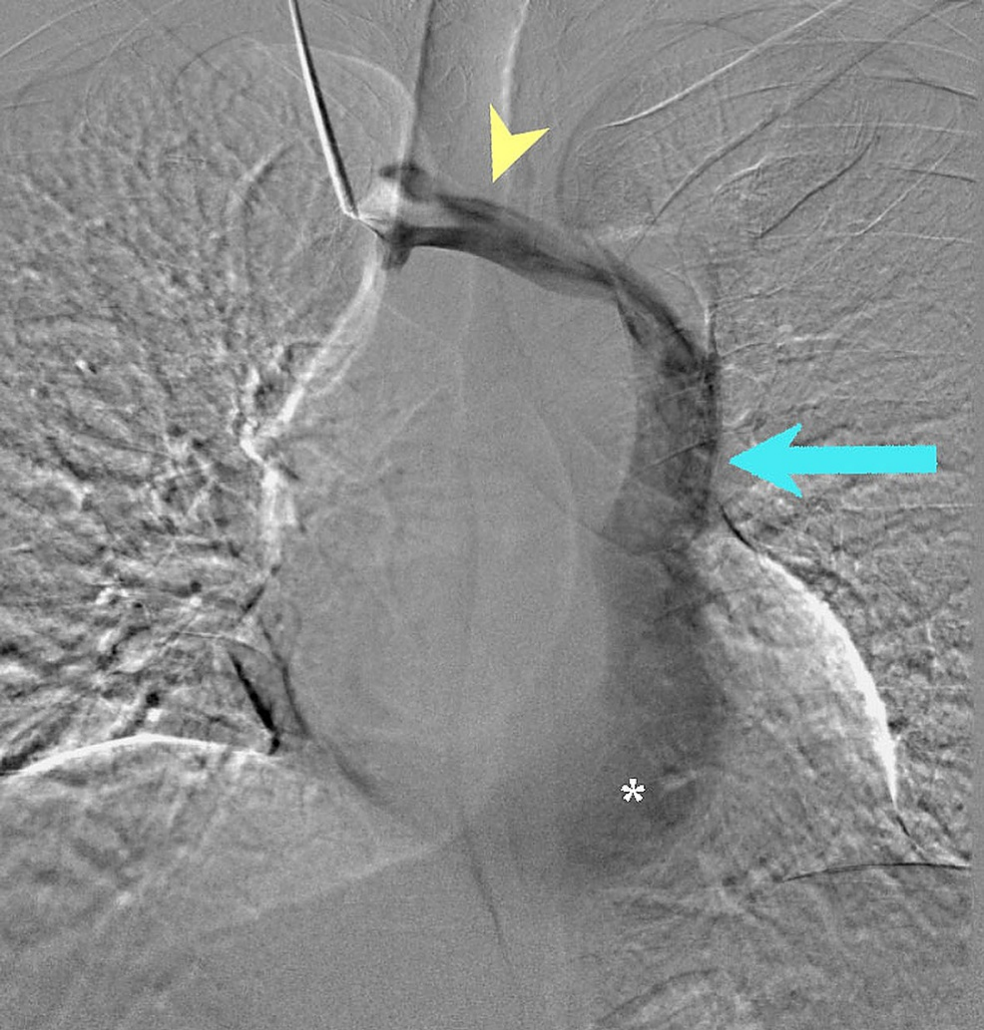
Fluoroscopic image upon contrast injection at the level of the right brachiocephalic vein. Fluoroscopic image upon contrast injection at the level of the right brachiocephalic vein showing the contrast filling the right brachiocephalic vein (arrowhead), which directly drains into the PLSVC (Arrow). The contrast continues into the dilated coronary sinus (*) without leakage to the left atrium.

A guidewire was passed, but it also went from the right jugular vein to the left thoracic cavity (Figure 3) (Video 2). Immediate contrasted venography was performed, in which contrast was injected at the level of the right brachiocephalic vein (Figure 4) that showed the flow of contrast through the right brachiocephalic vein to the left as it drained into the PLSVC. The contrast continued through the PLSVC into the dilated coronary sinus which drained into the right atrium, without contrast leakage into the left atrium, which made the possibility of the PLSVC drainage into the left atrium or an unroofed coronary sinus unlikely (Video 2) (Figure 4). An internal jugular line was inserted, and the patient underwent his first dialysis session for three hours. The patient was arranged to undergo femoral permacath insertion a week later, and cardiology was consulted to reconsider the possibility of arteriovenous fistula creation.

**Video 2:** Fluoroscopy video during catheterization upon contrast injection at the level of the right brachiocephalic vein showing the drainage to the isolated PLSVC then into the dilated coronary sinus (without any leakage into the left atrium) then into the right atrium.

**Figure 5:**
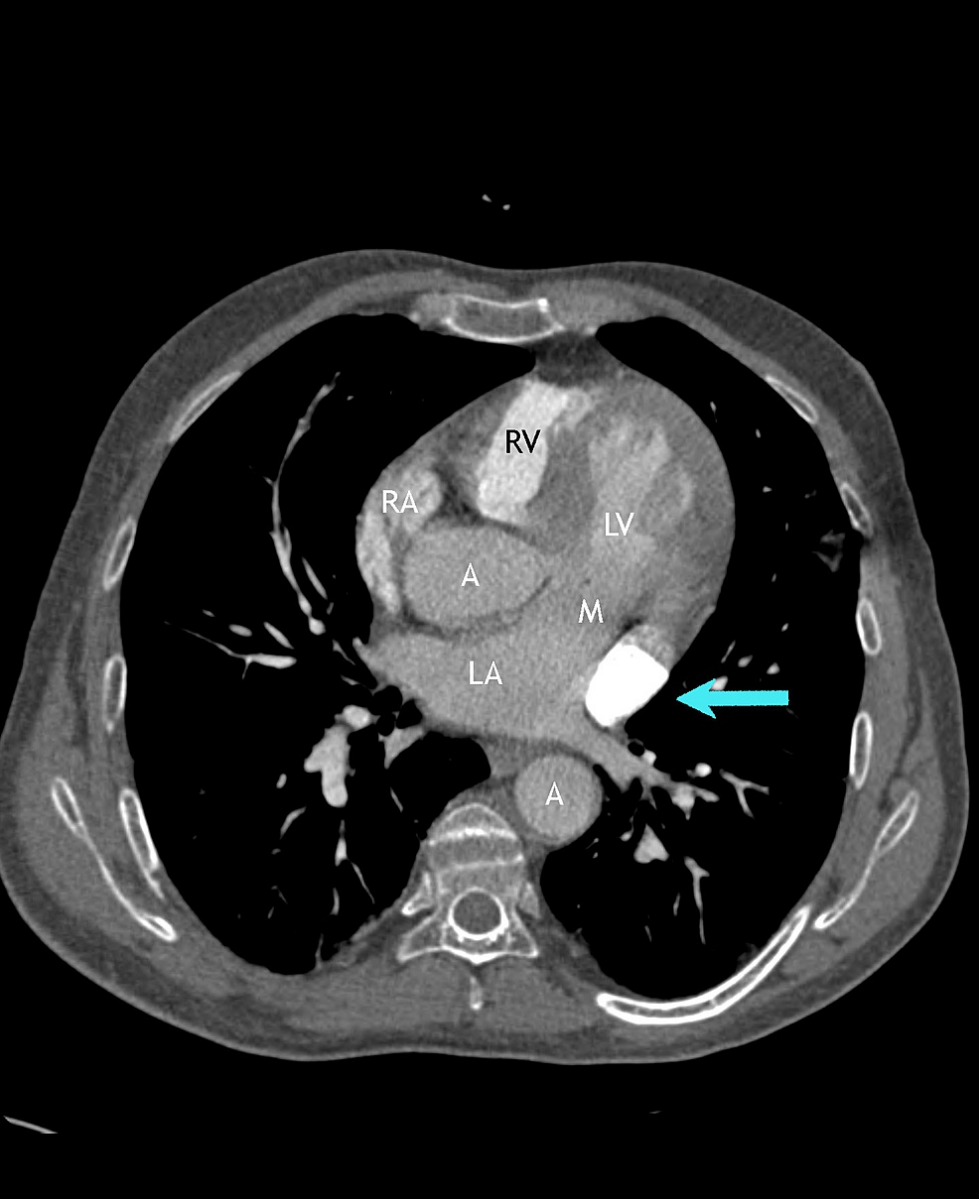
Axial CT angiogram following contrast injection in the right subclavian vein showing the isolated PLSVC (arrow) without evidence of any right-sided SVC draining into the normal position at the level of the right atrium LA: left atrium, LV: left ventricle, RA: right atrium, RV: right ventricle, M: mitral valve, A: aorta

Upon femoral permacath insertion, the right femoral vein was cannulated, and wire passed up to the IVC. However, substantial resistance was experienced, most probably due to the mass effect of polycystic kidneys. The patient's position was changed from a supine to a left lateral position which relieved the resistance. The catheter was flushed and ready to use. The patient was discharged from the hospital in good condition. CT angiogram following contrast injection in the right subclavian vein was performed showed an isolated PLSVC without evidence of any right-sided SVC draining into the normal position at the level of the right atrium (Figure 5).

**Figure 6:**
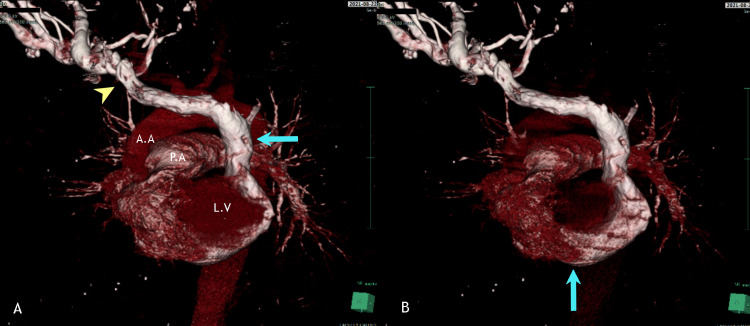
Anterio-lateral view of a 3D reconstruction of CT angiogram in which contrast was injected through the right subclavian vein. 6A: Anterio-lateral view of a 3D reconstruction of CT angiogram in which contrast was injected through the right subclavian vein. The right IJV and right subclavian drain into the right brachiocephalic vein (arrowhead), which then travels to the left side to drain into the isolated PLSVC (arrow). Notice absence of the normal right-sided SVC. 6B: After subtracting the appearance of the Left ventricle and aortic structures, the pathway of the isolated PLSVC is evident as it drains into the dilated coronary sinus (arrow), which follows a route inferio-posteriorly to the left ventricle. LV: left ventricle, AA: ascending aorta, PA: pulmonary artery

**Figure 7:**
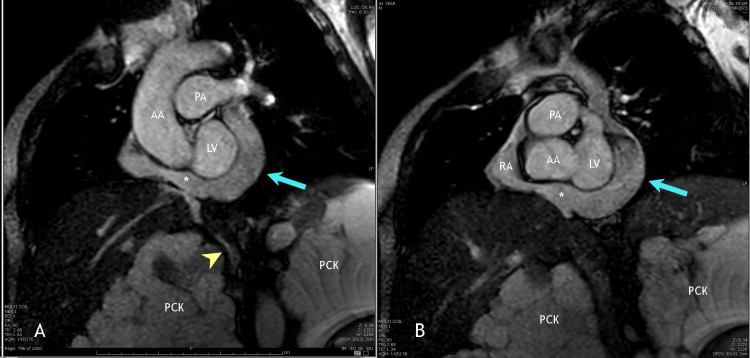
Coronal view of a cardiac MRI study (A) Coronal view of a cardiac MRI study showing the isolated PLSVC (arrow) as it drains into the dilated coronary sinus (*), which then drains into the atrial appendage. Notice the absence of any right-sided superior venous drainage. Also, notice the extremely enlarged polycystic kidneys (PCK), which are compressing the IVC (arrowhead). (B) Coronal view of a cardiac MRI study showing the previously mentioned path of the isolated PLSVC (arrow) into the dilated coronary sinus (*) then into the right atrial appendage (arrowhead). LV: left ventricle, RA: right atrium, AA: ascending aorta, PA: pulmonary artery, PCK: polycystic kidney

**Figure 8:**
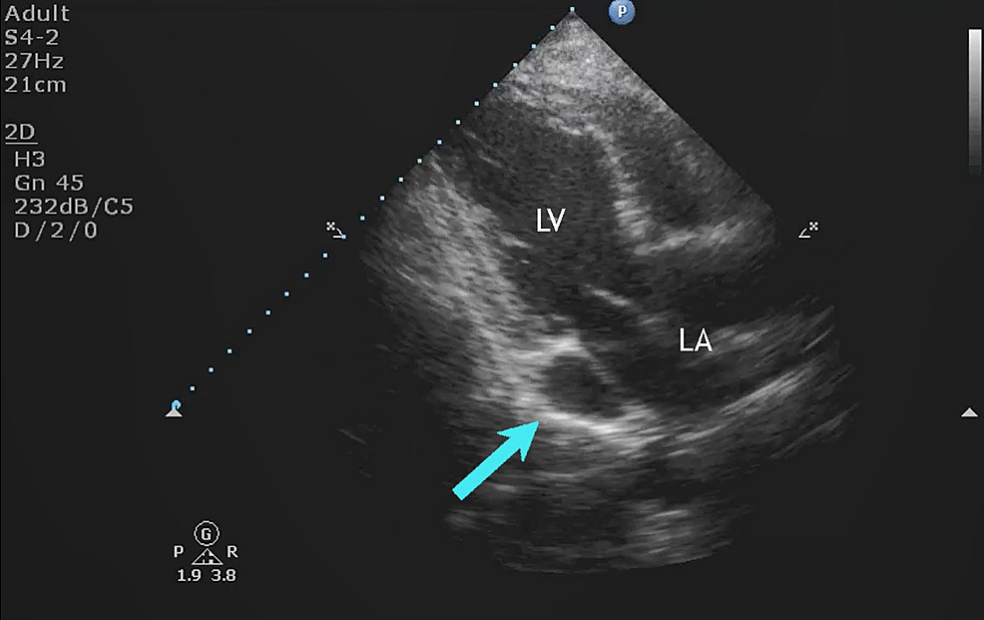
Echocardiogram showing the dilated coronary sinus. Echocardiogram showing the dilated coronary sinus (arrow). No associated congenital malformations were seen.

To further characterize the vascular anatomy of the patient and associated anomalies, a detailed cardiac MRI and 3D reconstruction technique of CT scans were performed. This showed the right brachiocephalic vein directly connected to the isolated PLSVC draining into the coronary sinus, which drained into the right atrium (Figures 6-7). It was also evident that the massive polycystic kidneys compressed the IVC, which drained into the right atrial appendage (Figure 7A). An echocardiogram showed the dilated coronary sinus, one of the cardinal signs of PLSVC. However, no congenital cardiac anomalies were found (Figure 8). The estimated LV ejection fraction was 30%. An EKG was done due to possible coexisting conduction abnormalities, which was unremarkable.

After making the diagnosis of PLSVC, we reviewed the patient’s records. The PLSVC was not documented, but it was noted in a previous chest CT scan done for another reason. Unfortunately, echocardiogram reports were missing. This emphasizes the importance of proper documentation of radiology reports on the patient electronic medical records, in addition to performing a thorough review of the patients' radiology reports before such complex interventions.

## Discussion

PLSVC is usually diagnosed during fetal life or as an incidental finding that is discovered after central venous catheterization is done for various reasons [1]. The catheter goes to the left mediastinum instead of the right, which should raise the suspicion of the presence of a vessel to the left side of the aorta [6]. Also, dilated coronary sinus and bubble injection techniques are helpful in the diagnosis of a PLSVC. Definitive diagnosis of PLSVC with absent RSVC is made by contrast imaging studies. Here, after the injection, of the contrast into the left arm vein, the contrast appears in the coronary sinus then into the right atrium [6].

There are various clinical correlations of PLSVC, including difficult central line catheterization, difficult pacemaker or defibrillator placement, and paradoxical thromboemboli through the commonly associated atrial septal defect. In addition, arrhythmias may result from the dilation of the coronary sinus opening that stretches the conduction system, especially if the coronary sinus is the only drainage from the SVC, like in our case [7]. Histologic evaluation of the conduction system in seven young patients with absent RSVC has been reported and showed hypoplastic SA and AV nodes [8].

According to Schummer's classification of the supracardial venous system, the anomaly in our case is classified as type 2 in which only a PLSVC exists, without the right superior vena cava [9,10] (Table 1). Also, according to Zhu's classification of PLSVC, our case is considered an isolated Type A PLSVC [10] (Table 2). Regarding the Internal Jugular Vein valves (IJVVs), previous literature reported that up to 90% of human IJVs contain similar valves [11]. IJVVs are the only barrier between the brain and the heart that prevents retrograde venous blood flow when the intrathoracic pressure increases. Malfunction of the IJVV may include restriction of the opening, which may impede insertion of the catheter or guidewire during central venous catheterization, or valve incompetence which may be a complication of catheterization [11].

**Table 1:** Schummer's classification of the supracardial venous system Schummer et al. [9] PLSVC: Persistent left superior vena cava

Types	Description
I	Normal superior vena cava anatomy
II	Only PLSVC exists, without the right superior vena cava (Isolated PLSVC)
IIIa	PLSVC and the right superior vena cava exist, with left brachiocephalic vein between both sides
IIIb	PLSVC and the right side of the superior vena cava, without left brachiocephalic vein between both sides

**Table 2:** Zhu’s classification of PLSVC Zhu [10]. PLSVC: Persistent left superior vena cava

Types	Description
A	PLSVC drains blood to the right atrium via the coronary sinus
B	PLSVC drains blood to the right atrium via coronary sinus with partial right-to-left shunt
C	PLSVC drains blood to the left atrium directly with a right-to-left shunt
D	PLSVC is directly connected to the left pulmonary vein

Another peculiar thing about our case was the coexistence of the persistent left SVC (PLSVC) with polycystic kidney disease. Careful literature review regarding the occurrence of renal anomalies with persistent left SVC revealed multiple cases of horseshoe kidney [12,13], unilateral absent kidney [14], and crossed fused ectopic kidney [15]. Co-occurrence of multicystic dysplastic kidney with PLSVC was reported four times: a case of familial 22q11.2 deletion syndrome [16], a case of complete trisomy 9 [17], a case of 17q12 deletion [18], and a case of polyalanine expansion mutation of ZIC3 gene [19]. Ichikawa et al. have compared the incidence and variations of superior vena cava (SVC) anomalies between patients with horseshoe kidneys and controls. He noted a higher incidence of anomalous SVC in patients with a horseshoe kidney than in controls, including one case of isolated PLSVC [12]. They also mentioned that the reasons for the coexistence remain unclear [12].

Besides horseshoe kidney, previously mentioned repetitive renal anomalies may point towards an association between PLSVC and renal anomalies despite being heterogenous in pathogenesis. The coexistence of renal anomalies and PLSVC as part of genetic disorders could support an association. To our knowledge, our case is distinguished by being the first to report autosomal dominant polycystic kidney disease as a coexistent renal anomaly with isolated PLSVC.

## Conclusions

To conclude, we suggest performing an echocardiogram on known renal congenital disease patients to look for various co-occurring cardiac and vascular malformations. Also, interventional radiologists, electrophysiologists, and intensivists should keep the possibility of PLSVC in mind, especially when difficulties are encountered during catheterization where proper characterization of the PLSVC type and associated anomalies is crucial for tailoring the best management approach.
